# Geospatial analysis of human-elephant conflict zones in the Jeli District: implications for mitigation and land-use planning

**DOI:** 10.1007/s10661-025-14967-7

**Published:** 2026-01-14

**Authors:** Hazizi Husain, Amal Najihah Muhamad Nor, Muhamad Azahar Abas, Aainaa Amir, Nur Hairunnisa Rafaai, Siti Balqis Jaafar, Ashiah Rosdi, Farah Nabila Ahmad, Fazrin Munirah Atan, Ahmad Shahdan Kasim, Hairulazim Mahmud, Salman Saaban, Kamarul Hambali

**Affiliations:** 1 Faculty of Earth Science, Universiti Malaysia Kelantan, Jeli Campus, 17600 Jeli, Kelantan Malaysia; 2 Jabatan PERHILITAN Negeri Kelantan, Tingkat 12 Wisma Persekutuan Jalan Bayam, 15664 Kota Bharu, Kelantan Malaysia; 3 Environment and Sustainable Development Research Group, Universiti Malaysia Kelantan, Jeli Campus, 17600 Jeli, Kelantan Malaysia; 4 UMK-Tropical Rainforest Research Centre (UMK-TRaCe), Tasik Banding, Royal Belum, 33300 Gerik, Perak Malaysia; 5 Animal and Wildlife Research Group, Faculty of Earth Science, Universiti Malaysia Kelantan, Jeli Campus, 17600 Jeli, Kelantan Malaysia; 6 Institute for Environment and Development (LESTARI), Universiti Kebangsaan Malaysia (UKM), 43600 Bangi, Selangor Malaysia; 7 Malaysian Palm Oil Green Conservation Foundation (MPOGCF), Level 12-3-3A, PJX HM Shah Tower, No. 16 A, Jalan Persiaran Barat PJS 52, 46200 Petaling Jaya, Selangor Malaysia; 8 Jabatan PERHILITAN, KM. 10, Jalan Cheras, 56100 Kuala Lumpur, Malaysia

**Keywords:** Human-elephant conflict (HEC), Habitat fragmentation, Geographic Information System (GIS), Hotspot analysis, Land-use planning, Wildlife-human coexistence

## Abstract

Human-elephant conflict (HEC) is a growing challenge in the Jeli District, largely driven by habitat fragmentation due to agricultural expansion, human settlements, and changing land use patterns. As elephants are pushed out of their natural habitats, they increasingly come into contact with human populations, especially in agricultural areas such as oil palm plantations. These encounters often result in crop damage, economic losses, and safety concerns for local communities. The conflict is intensified by the loss of continuous forest cover and the limited availability of safe, natural movement routes for elephants. This study examines the spatial patterns and drivers of HEC in Jeli using Geographic Information System (GIS) tools, including distribution mapping and hotspot analysis, to identify areas with high conflict intensity. The findings highlight critical zones where human and elephant activities overlap, providing essential insights for land-use planning, conflict mitigation strategies, and the development of long-term coexistence solutions between rural communities and wildlife.

## Introduction

Habitat loss and fragmentation are among the leading drivers of global biodiversity decline, particularly in tropical regions where deforestation rates remain exceptionally high (Burgess et al., 2002; Sodhi et al., 2010). In tropical Asia, rapid land-use change driven by agriculture expansion, commercial logging, infrastructure development, and urbanization has led to extensive losses of primary forest (Achard et al., 2002; Laurance et al., 2000). Recent global forest cover assessments show that Southeast Asia continues to experience some of the world’s highest deforestation rates, posing serious consequences for wildlife species that rely on large, continuous habitats (Curtis et al., 2018; Nad et al., 2022). In Peninsular Malaysia, forest cover decreased from 6.2 million hectares in 1990 to 5.7 million hectares in 2018, with lowland dipterocarp forests being disproportionately affected due to their accessibility and high commercial value (Kinnaird et al., 2003). As forest ecosystems become increasingly fragmented, connectivity declines, thereby compromising ecological processes essential for long-term biodiversity persistence (Fahrig, 2003; Hadded et al., 2015).

These landscape changes have severe implications for wide-ranging mammals such as the Asian elephant (*Elephas maximus*), which is currently listed as Endangered on the IUCN Red List (Williams et al., 2020). Elephants require expansive, connected habitats to fulfil their ecological needs, with home ranges often spanning hundreds of square kilometers (Lynam et al., 2007; Sukumar, 2003). Across many Asian range countries, including India, Sri Lanka, Thailand, Myanmar, and Malaysia, elephants are increasingly forced into agricultural and peri-urban areas as a result of habitat loss (Fernando et al., 2005; Sukumar, 2006). Consequently, human-elephant conflict (HEC) has escalated over recent decades, leading to crop damage, property loss, and both human and elephant casualties (Naha et al., 2019; Naughton-Treves et al., 1998). This reflects a broader global pattern where habitat fragmentation intensifies human-wildlife interactions and reduces landscape capacity to support large mammals (Ripple et al., 2015; Woodroffe & Frank, 2005).

In Malaysia, HEC represents a major conservation and socio-economic challenge, particularly in states undergoing rapid agricultural expansion and infrastructure development. Between 2013 and 2019, the Department of Wildlife and National Parks (PERHILITAN) documented 2,930 HEC complaints, with financial losses exceeding RM31 million (PERHILITAN, 2020). Johor, Pahang, Perak, Kelantan, Terengganu, and Kedah record the highest number of incidents (Saaban et al., 2011). Kelantan has experienced especially severe conflict due to extensive forest clearing for rubber, oil palm, and settlement schemes, with 17 elephant-related attacks reported between 2013 and 2019, resulting in 10 injuries, 7 fatalities, and the capture of 37 conflict elephants (PERHILITAN, 2020). The Jeli District, located along a major forest-agriculture interface, is consistently identified as one of the most significant conflict hotspots in Peninsular Malaysia (Magintan et al., 2012). As fragmentation intensifies, forest edges become increasingly permeable, enabling elephants to move more easily into villages and plantations (Leimgruber et al., 2003).

Despite the seriousness of HEC in Malaysia, spatially explicit research on elephant movement and conflict hotspots remains limited. While several Asian countries have employed GIS-based modeling and species distribution approaches to identify conflict-prone areas, similar studies in Malaysia are scarce and geographically restricted (Fernando et al., 2008; Goswami & Vasudev, 2017). Such modeling tools are vital for informing national conservation priorities, guiding land-use planning, and supporting ecological corridor development under Malaysia’s National Policy on Biological Diversity (NPBD 2016–2025). Predictive tools such as MaxEnt, which operate using presence-only data, are particularly valuable in data-limited contexts and have demonstrated strong performance in wildlife conflict and habitat suitability analyses across Asia (Phillips, Dudík & Schapire 2004; Brotons et al., 2004; Lurance, Goosem & Laurance 2009).

A wide range of species distribution modeling (SDM) approaches is available, including generalized linear models (GLMs), generalized additive models (GAMs), random forests, boosted regression trees (BRT), and ecological niche factor analysis (ENFA) (Elith et al., 2006; Franklin, 2010). However, many of these methods require extensive presence-absence datasets or large sample sizes, which are often lacking for elusive species such as elephants or for conflict reports that are typically collected opportunistically. In contrast, the Maximum Entropy (MaxEnt) model (Phillips, Dudík & Schapire, 2004) is specifically designed for presence-only data, making it highly suitable for HEC research where confirmed conflict locations exist but absence data are unreliable. MaxEnt has shown strong predictive performance under small sample sizes and in heterogeneous landscapes (Elith et al., 2011).

Due to its ability to handle incomplete datasets, MaxEnt has become one of the most widely used tools for modeling species distributions and human-wildlife conflict risk worldwide (Merow et al., 2013). These models support evidence-based decision-making by informing corridor design, land-use planning, and community-based conflict mitigation strategies (Brotons et al., 2004; Laurance, Goosem, and Laurance 2009). As such, MaxEnt represents an appropriate and effective choice for predicting HEC risk in fragmented landscapes like the Jeli District, where presence-absence data are limited but conflict occurrence records are available.

Although several studies in Malaysia have examined HEC patterns at broader state or national scales (Saaban et al., 2011), fine-scale spatial analyses in key conflict hotspots such as Kelantan remain scarce. Most previous work has focused on descriptive assessments, community perceptions, or general conflict trends, with limited application of predictive spatial modeling to identify emerging high-risk zones (Ahmad Zafir & Magintan, 2016; Jamaluddin et al., 2024). In contrast, countries such as India, Sri Lanka, and Nepal increasingly incorporate GIS-based SDMs to understand conflict risk, yet comparable high-resolution analyses in Malaysia remain sparse and localized (Fernando et al., 2008; Goswami & Vasudev, 2017). This lack of locality-specific predictive mapping constrains the ability of wildlife authorities to implement targeted mitigation strategies in rapidly changing landscapes such as the Jeli District. In response to these gaps, this study aims to (i) identify the spatial distribution and hotspots of human-elephant conflict in the Jeli District of Kelantan, and (ii) predict potential future conflict zones using spatial modeling approaches. These insights are intended to support proactive conservation planning, strengthen coexistence strategies, and reduce the socio-economic impacts of HEC on affected communities.

## Materials and methods

### Study area

**Fig. 1:**
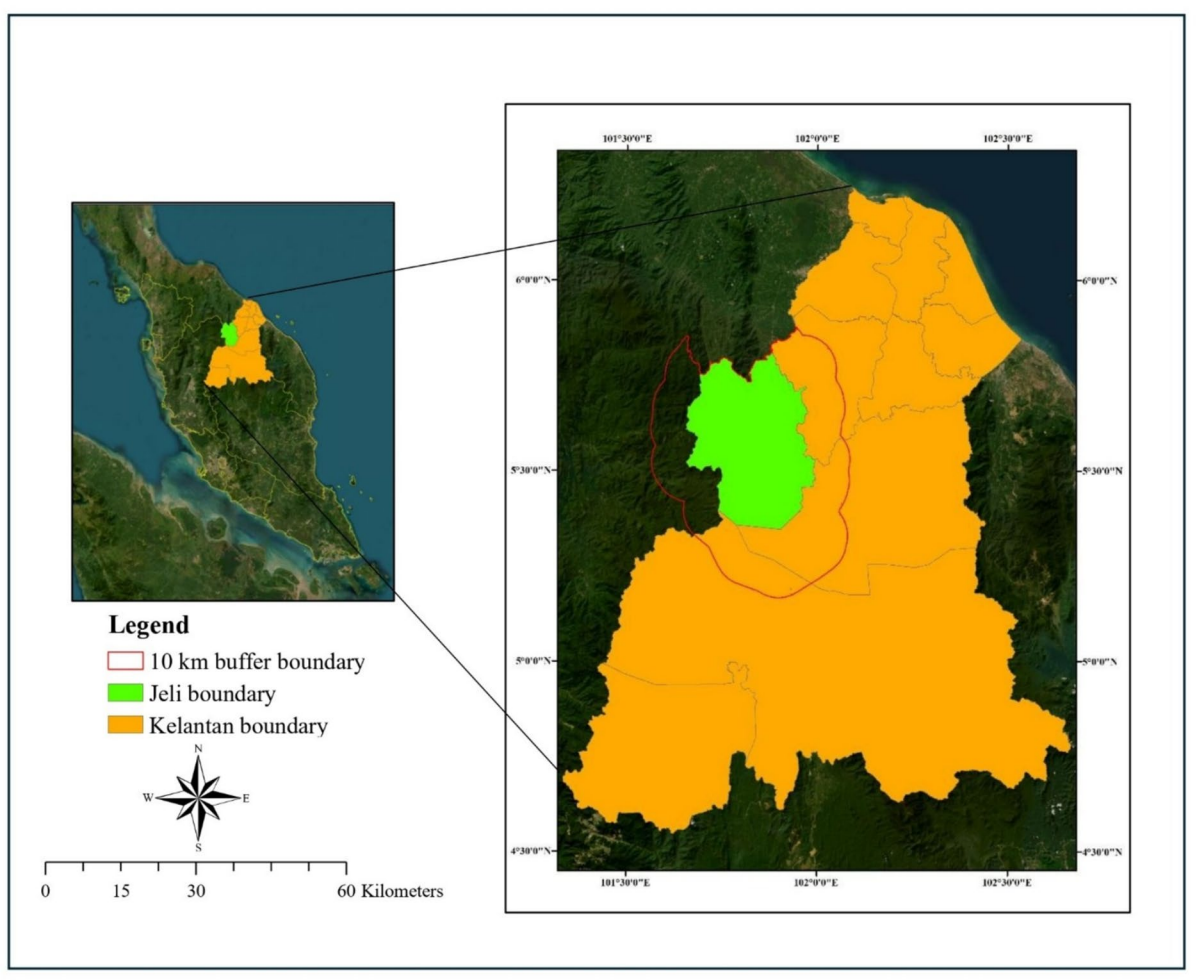
Map of Jeli District, Kelantan, Peninsular Malaysia (creator: Nur Hairunnisa Rafaai, software ArcGIS 10.8)

The study was conducted in the Jeli District of Kelantan, Peninsular Malaysia (5.7007° N, 101.8432° E), covering an area of approximately 1333 km^2^ (Fig. 1). Located in the northwestern part of Kelantan, Jeli borders Thailand to the north and the Titiwangsa Range to the west. The district is administratively divided into three subdistricts: Batu Melintang, Jeli, and Kuala Balah, and had a population of 39,710 in 2010, with 43.78% engaged in agricultural activities (DOSM 2010).

The landscape of Jeli is predominantly hilly, with elevations ranging from 150 m to 1840 m above sea level. It is traversed by five major rivers: Pergau, Renyuk, Suda, Terang, and Balah. Forest and vegetation constituted a major portion of the Jeli District land use, with forest cover remaining significant despite changes due to development between 1984 and 2012 (Abdul Karim et al., 2020). Of this, approximately 47.34% is managed by the Kelantan State Forestry Department. Despite this, parts of the forest have experienced repeated logging since the 1970s, with several areas still under active timber extraction (Rayan & Mohamad, 2009). Land use change analysis in the Jeli District shows a significant expansion of agricultural areas, particularly rubber and oil palm plantations, alongside reduction in forest cover, reflecting that agriculture constitutes a major land-use category in the district (Abdul Karim et al., 2020).

In general, the average annual minimum temperature in Kelantan is approximately 23.4 °C and the maximum temperature is 32.3 °C (DOSM 2023). In 2022, the region experienced an estimated rainfall ranging from 3220.0 mm to 4508.6 mm (DOSM 2023). According to METMalaysia, peak rainfall is usually observed during the northeast monsoon, which occurs from November to January, with additional peaks in April and May. On the other hand, June and July are identified as the driest months. These variations in temperature and rainfall affect the movement patterns of elephants, especially during prolonged dry periods when they migrate to human settlements in search of water sources. In Jeli, the main land use and land cover in 2022 consisted of 65.48% forest, 30.56% agriculture, and 1.54% water bodies (PLANMalaysia 2024). Land use changes, particularly the expansion of smallholder oil palm, rubber, and fruit plantations, including bananas, corn, and various fruit trees such as durian, jackfruit, rambutan, and papaya, have contributed to the emergence of high-attraction zones for elephants. In addition, human-elephant conflict (HEC) usually peaks during the dry season, as elephants concentrate their activities in agricultural areas. The combined effect of these factors has led to an increase in human-elephant conflict in this district.

Ecologically, Jeli forms part of a crucial wildlife corridor linking the Titiwangsa Range to the Belum-Temengor Forest Complex (BTFC), a nationally significant conservation area. The region supports a diverse range of fauna, including the Asian elephant (*Elephas maximus*), Malayan tiger (*Panthera tigris jacksoni*), tapir (*Tapirus indicus*), and other threatened species such as the gaur (*Bos gaurus*), siamang (*Symphalangus syndactylus*), and dhole (*Cuon alpinus*) (WWF-Malaysia, 1991). Bamboo, a key food source for elephants, is particularly abundant in the northeastern forests of Jeli and Pergau (Pue & Latiff, 2005).

### Elephant conflict analysis

Human-elephant conflict (HEC) is intensifying due to extensive land-use change, agricultural expansion, and the fragmentation of natural habitats. In Jeli District, Kelantan, this study investigates the spatial and temporal dynamics of HEC to identify underlying drivers, spatial patterns, and conflict hotspots. Conflict data from 2015 to 2023 were analyzed using the Maxent model (Phillips et al., 2006), a presence-only modeling approach effective in predicting habitat suitability and potential conflict zones based on environmental variables.

MaxEnt modeling facilitates the identification of high-risk areas by estimating the probability distribution of species occurrence based on environmental predictors. In this study, MaxEnt was run using a suite of ecologically relevant covariates that influence elephant movement and conflict patterns. The predictor variables included collared elephant distribution, land-use/land-cover, NDVI, elevation, slope, distance to the river, and distance to the roads. All covariates were resampled to a uniform spatial resolution and checked for multicollinearity using a variance inflation factor (VIF) threshold (e.g., VIF < 10).

Model performance was evaluated using the Area Under the Receiver Operating Characteristic Curve (AUC/ROC), which provides a threshold-independent measure of model accuracy. The MaxEnt model in this study achieved an AUC value of 79.4%, indicating high accuracy and good predictive performance. Higher AUC values reflect a greater ability of the model to discriminate between presence locations and background points.

MaxEnt analyzes the presence-only data by estimating the probability distribution of species occurrence that is closest to uniform (i.e., maximum entropy) while still being constrained by the environmental conditions at known presence locations. The model outputs include a habitat suitability map, response curves, permutation importance, and Jackknife tests of variable importance, all of which help identify the environmental factors most strongly associated with elephant occurrence. The resulting suitability map highlights potential conflict hotspots, supporting targeted mitigation strategies such as wildlife corridor planning, installation of electric fences, and improved land-use zoning in high-risk areas.

Complementary to this, movement data from GPS-collared elephants, Mek Pergau (collared in 2012) and Pak Mat Pauh (collared in 2022), were obtained from the Department of Wildlife and National Parks (PERHILITAN) Kelantan. The collaring procedures were conducted exclusively by PERHILITAN’s trained wildlife veterinarians and field biologists as part of the department’s long-term elephant monitoring program. Animals were immobilized following standard veterinary protocols using approved anesthetic agents, after which GPS collars were fitted securely and adjusted to ensure proper movement and prevent chafing.

All collaring activities were carried out under official permits and ethical approval issued by PERHILITAN, adhering to the Wildlife Conservation Act 2010 (Act 716) and PERHILITAN’s Standard Operation Procedures (SOPs) for Wildlife Capture and Handling. Continuous monitoring during immobilization and recovery ensured minimal stress to the animals, and both elephants resumed normal activity shortly after release. No adverse effects, injuries, or long-term behavioral changes associated with collaring were reported by PERHILITAN.

The collars recorded spatial positions every four hours over approximately a 12-month period, providing high-resolution movement data for ecological and conflict risk analyses. The dataset, provided in XML format and later converted for geospatial analysis, included latitude, longitude, elevation, and reconstructed movement trajectories.

Integration of GPS telemetry data with conflict occurrence improves the spatial resolution of conflict prediction models and enhances understanding of elephant behavior in fragmented landscapes. This integrated approach is critical for identifying key conflict zones and informing proactive, evidence-based mitigation strategies in Jeli and similar human-wildlife conflict-prone areas.

### Hotspot analysis

In the context of human-elephant conflict (HEC), hotspot analysis refers to the identification and spatial mapping of areas with the highest concentration of conflict incidents. This approach employs geospatial data to predict and delineate locations where conflict events are most likely to occur, providing a strategic foundation for targeted mitigation.

A widely used spatial statistical method for hotspot detection is the “Getis-Ord Gi” statistic, which evaluates the degree of spatial clustering by computing z-scores for each feature to determine whether it forms part of a statistically significant concentration of high or low values (Getis & Ord, 1992; Ord & Getis, 1995). Positive z-scores identify hotspots where high-intensity events cluster, whereas negative z-scores indicate cold spots. Higher positive z-scores reflect stronger spatial aggregation of conflict incidents and therefore help pinpoint critical zones of elephant-human interaction.

In this study, spatial records of elephant conflict events served as the primary input for hotspot analysis, and the “Getis-Ord Gi” index was calculated to detect locations where the observed intensity of incidents differed significantly from what would be expected under a random spatial distribution. This approach allowed the identification of persistent risk areas that can inform targeted management actions and support proactive mitigation strategies. The “Getis-Ord Gi” index is calculated as follows:where

Gᵢ*: Index value for location i.
xⱼ: Attribute value of location j.
wᵢⱼ: Multiplication factor between locations i and j.
X: Average attribute value.
S: The standard set of attribute values.
n: Number of locations.

To further visualize the spatial distribution of conflict intensity, Inverse Distance Weighting (IDW) interpolation was applied. IDW generates a continuous surface map based on the principle that nearby points have more influence on predicted values than those farther away, producing a spatially smooth representation of HEC intensity.

Geographic Information Systems (GIS) software, including ArcGIS desktop 10.8 (Gorr & Kurland, 2020) and QGIS Desktop 3.44.5 (QGIS Development Team, 2024), were used to map elephant population distribution, conflict locations, and identified hotspots. Spatial analyses such as the Minimum Convex Polygon (MCP) were performed to delineate elephant home ranges and primary movement corridors (Hayne, 1949; Mohr, 1947). By overlaying movement zones with “Getis-Ord Gi” derived conflict hotspots, the study was able to identify high-risk intersection areas, thereby improving the spatial precision and effectiveness of conflict mitigation planning.

By integrating spatial statistical analysis with ecological and geospatial tools, this study provides a robust framework for identifying critical conflict zones, guiding conservation interventions, and supporting landscape-level coexistence strategies between humans and elephants.

**Fig. 2:**
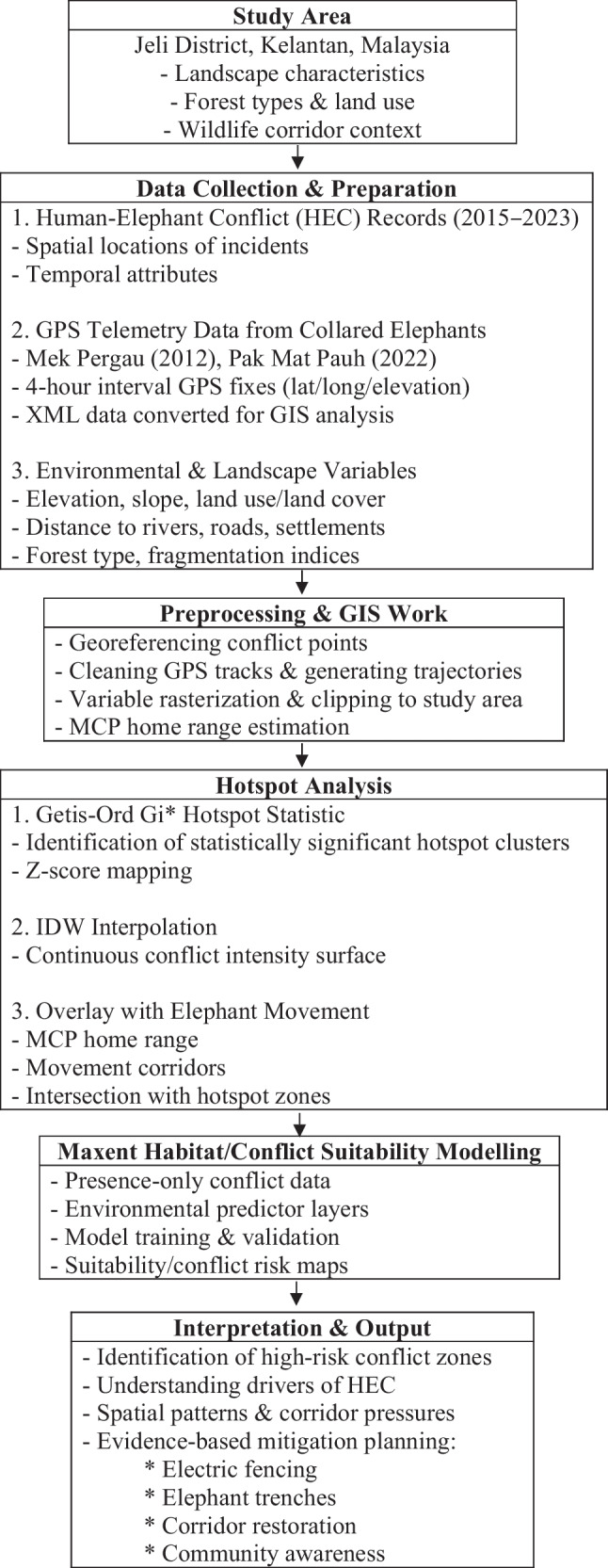
Methodological flow chart illustrating the integrated framework used to assess human-elephant conflict (HEC) dynamics in Jeli District, Kelantan

The overall sequence of analytical steps used in this study is summarized in Fig. 2. The flow chart outlines how the various datasets and spatial analyses were brought together to assess conflict patterns and identify areas of elevated risk.

## Results

### Hotspot analysis

Table 1 shows hotspots with high elephant activity based on color. Elephant hotspot areas have been identified mainly in the North-West and South-West of the Jeli District. Areas near forest reserves and agricultural areas are frequently subject to elephant encroachment. These hotspots are areas where elephant presence is high and where human-elephant conflict often occurs. To ensure that Jeli elephants have sufficient space to move and forage, their management and conservation require a holistic approach that involves reducing conflict, protecting their habitat, and monitoring their distribution and movements. This study aims to identify hotspots of elephant-human interaction and determine the need to create elephant corridors to reduce human-elephant conflict. Elephant management and conservation in the Jeli District will be more effective and sustainable in the long term if these strategies are combined.

**Table 1:** Interpretation of *Z*-score range

Color	*Z*-score range	Interpretation
Red	3.932 to 5.421	Hotspots are very significant
Orange	2.149 to 3.931	Significant hotspots
Yellow	0.843 to 2.148	Moderately warm areas
Light green	−0.220 to 0.843	Low attendance
Green	−0.619 to −0.220	No activity

**Fig. 3:**
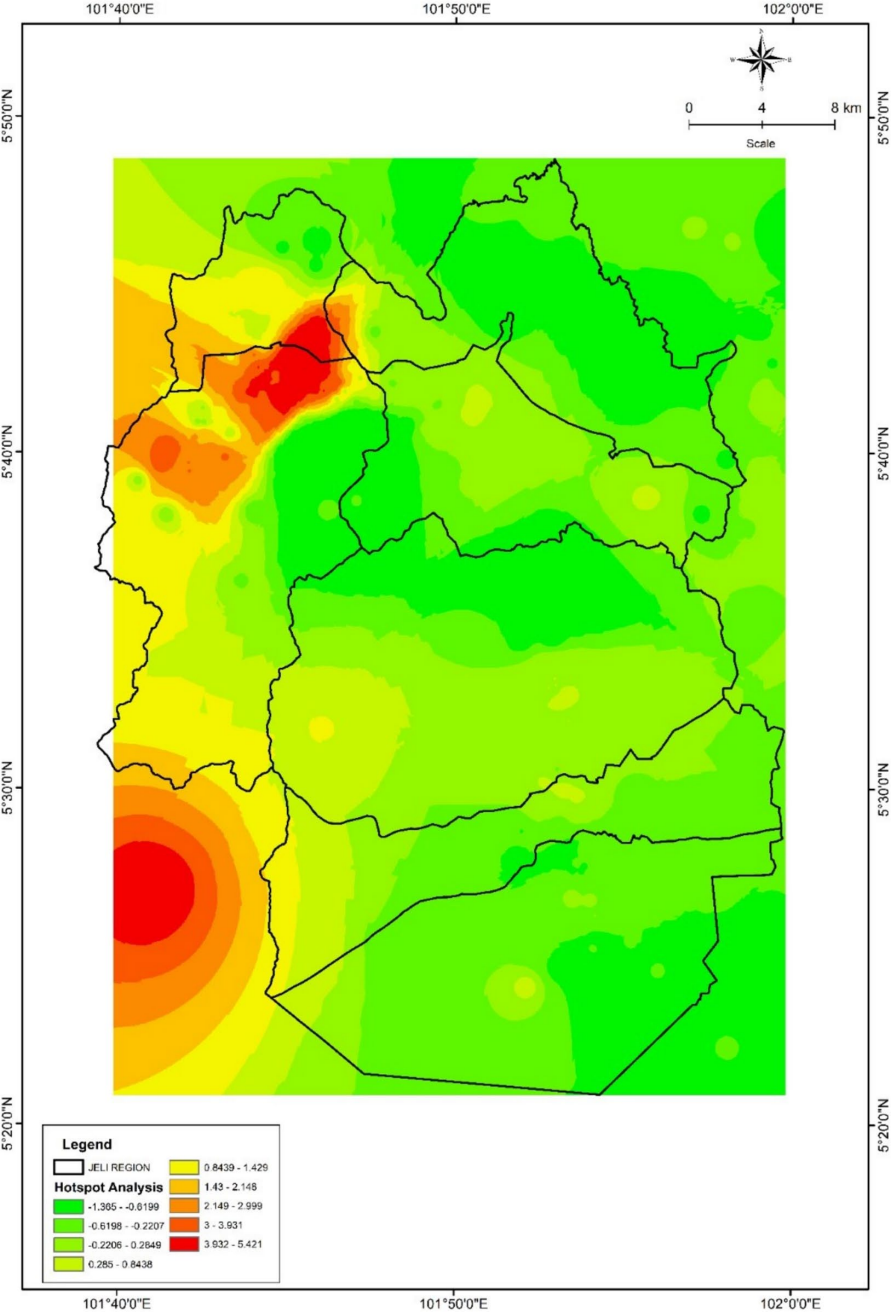
Hotspot analysis (creator: Amal Najihah Muhamad Nor, software ArcGIS ver 10.8)

Figure 3 shows hotspots where red indicates the highest hotspots while green indicates areas with fewer hotspots. Elephant hotspots are usually located near their original habitat, namely areas near forest reserves, national parks, and wildlife corridors, agricultural areas that attract food for elephants such as oil palm, rubber, banana, and rice plantations, densely populated areas, and elephant migration corridors, which are natural routes that elephants often use to move from one area to another.

### Elephant conflict simulation

**Fig. 4:**
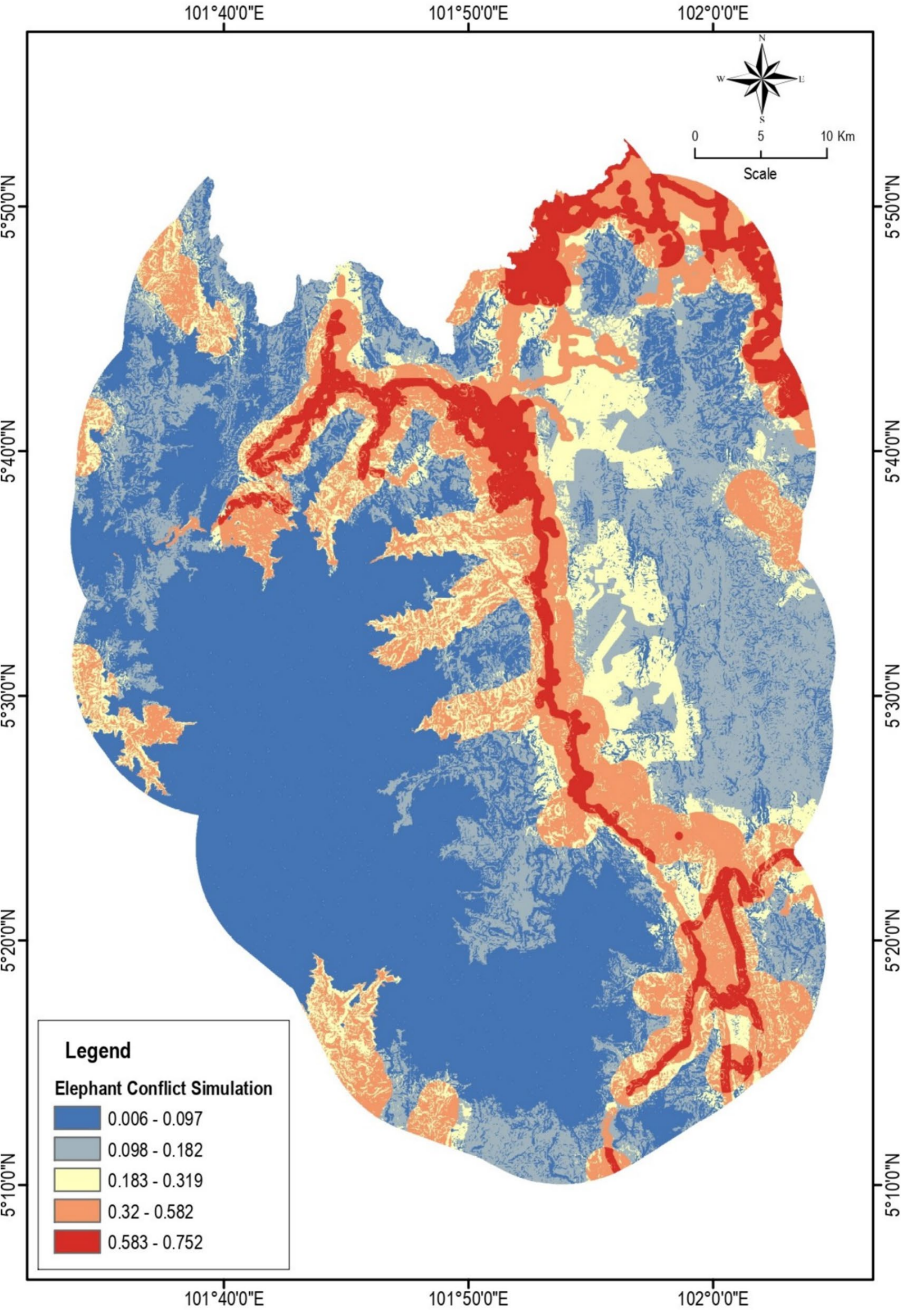
Elephant conflict simulation map (creator: Nur Hairunnisa Rafaai, software MaxEnt ver 3.4.3)

Elephant conflict simulation was conducted using Maxent software for elephant conflict from 2015 to 2023. Figure 4 shows the results of the elephant conflict simulation in the Jeli District based on the overlay analysis of various spatial factors, where red color indicates potential for elephant conflict while yellow color indicates areas with less elephant conflict. The increase in conflict complaints increased over the 10-year period, mostly concentrated in road areas, forest edges with agriculture, and human settlements close to forests. On the map, colors from low values (dark blue) to high values (dark red) indicate the level of potential conflict between elephants and humans (Table 2).

**Table 2:** Interpretation of colors and conflict classes

Color	Value	Class conflict
Dark blue	0.000–0.097	Areas with a very low probability of conflict. These areas usually consist of forests far from human settlements
Light blue–yellow	0.098–0.319	Transition zone between natural habitats and areas of human activity such as settlements and agriculture
Orange–red	0.32–0.752	Moderate to high conflict zones. Areas close to roads, villages, farms and development areas that encroach on elephants’ original habitat

Overlay pattern:

- The prominent red lines indicate elephant movement corridors that connect to areas of development and human activity, such as major roads and agricultural areas.
- This overlay analysis shows that areas at high risk for conflict are mainly in the central and eastern parts of the Jeli District.
- Areas to the southwest and northwest of Jeli are still less likely to be used as buffer zones or habitat conservation.

The overlay analysis in this map shows how the combination of various spatial factors. This information is important for defining conflict mitigation zones, planning wildlife corridors, and supporting sustainable land use management decisions by local authorities and PERHILITAN.

## Discussion

Hotspot analysis underscores the spatial concentration of HEC in northwestern and southwestern zones of Jeli, particularly in areas bordering forest reserves and plantation lands. These regions correspond with known elephant habitats and foraging grounds, including areas rich in bamboo and lowland dipterocarp species (Pue & Latiff, 2005), which attract elephants into agricultural zones. The clustering of conflict incidents in proximity to such ecologically and economically significant zones suggests a pressing need for integrated landscape planning that balances conservation goals with agricultural productivity. As pointed out by Brotons et al. (2005) and Nad et al. (2022), habitat mosaics where natural and human-modified environments intersect often become focal points for conflict, thus requiring spatially explicit management interventions. These may include the establishment of buffer zones, seasonal deterrent strategies, and community engagement programs tailored to hotspot zones.

The conflict simulation results reveal that spatial overlap between elephant movement routes and human land use is a primary driver of conflict intensity in Jeli. The central and eastern parts of the district, which have undergone significant agricultural conversion and infrastructure expansion, correspond to areas marked in red on the conflict prediction maps. These areas are highly susceptible to elephant intrusions due to the loss of continuous forest cover and proximity to key movement corridors. This finding is consistent with earlier research by Leimgruber et al. (2003) and Fernando et al. (2005), which emphasized that habitat fragmentation and corridor disruption force elephants to move through human-dominated landscapes, thereby escalating conflict risks.

The integration of GPS telemetry data from collared elephants, Mek Pergau and Pak Mat Pauh, supports the simulated predictions by confirming frequent elephant movements along forest-agriculture boundaries and near road networks. The overlap between high GPS activity and the red-zoned conflict areas indicates that elephants are persistently utilizing these contested spaces as part of their movement pathways. This reinforces findings by Laurance et al. 2009, who highlighted the ecological implications of linear infrastructure development in tropical regions, noting that roads and clearings act as both barriers and attractants for large mammals. Moreover, the telemetry data suggest elephants are adapting to anthropogenic pressures by altering their movement timing and routes, which complicates mitigation strategies and demands dynamic corridor design.

## Conclusion

This study highlights the growing intensity of human-elephant conflict (HEC) in the Jeli District of Kelantan, primarily driven by habitat fragmentation, agricultural expansion, and increasing human encroachment into elephant habitats. Using Geographic Information System (GIS) tools, Maxent modeling, and hotspot analysis, we successfully identified key conflict zones and high-risk areas where elephant movement overlaps with human activity, particularly near forest edges, roads, and agricultural lands. The spatial analysis revealed that the central and eastern parts of Jeli, which are close to settlements and plantations, experience the highest levels of conflict. GPS telemetry data from collared elephants further confirmed that elephants frequently traverse these human-dominated landscapes, emphasizing the need for functional and protected movement corridors. The hotspot analysis, based on “Getis-Ord Gi” statistics and IDW interpolation, provided fine-scale insights into zones with significant elephant activity and recurring conflict. These findings underscore the urgent need for integrated land-use planning, targeted conflict mitigation strategies, and community-based awareness programs. The establishment of ecological corridors, enforcement of buffer zones, and sustainable land management practices are essential to reduce conflict intensity and ensure long-term coexistence between humans and elephants. By leveraging spatial tools and ecological data, this study provides a data-driven foundation for wildlife conservation and conflict mitigation in Jeli and other regions facing similar challenges.

## Data Availability

No datasets were generated or analyzed during the current study.
